# “God is in Control”: Religious Coping in Sermons About the COVID-19 Pandemic in the Reformed Church in Zambia

**DOI:** 10.1007/s10943-023-01915-3

**Published:** 2023-09-27

**Authors:** Johanneke Kroesbergen-Kamps

**Affiliations:** 1 Department of Religion Studies, Faculty of Theology and Religion, University of Pretoria, Pretoria, South Africa; 2 Department of History, European Studies & Religious Studies, Faculty of Humanities, University of Amsterdam, Amsterdam, The Netherlands

**Keywords:** Religious coping, COVID-19, African Christianity, Zambia

## Abstract

Coping is one of the mechanisms employed by people to deal with crises or disasters such as the global COVID-19 pandemic. The RCOPE, developed by Kenneth Pargament et al., is a quantitative scale to measure styles of religious coping. This article applies the RCOPE qualitatively to live-streamed sermons in the Reformed Church in Zambia, held in the two-month lockdown period in Zambia from the end of March to the end of May 2020. A total of 20 pastors contributed 134 sermons that were transcribed and analyzed using the full RCOPE. The results show that pastors in the RCZ mainly encourage their audiences to seek spiritual support, gain control over the situation, and give a positive meaning to the pandemic. The idea that God is in control is important behind all of these means of religious coping in the Zambian sermons. This qualitative analysis also discovered possible lacunae in the RCOPE questionnaire, especially concerning its applicability to evangelical forms of global Christianity, such as the lack of attention to God’s intervention and control.

## Introduction

The global COVID-19 pandemic was much more than a health crisis. It affected economies; people’s businesses, and their ability to work and earn money. It also affected the social fabric through restrictions on interactions between people, arguments and conflicts about these regulations, and the stigma that came with a possible infection. The pandemic was a disaster that even those whose health is unaffected needed to find ways to deal with. In psychology, the process of using strategies and efforts to manage the demands of a taxing and stressful situation, such as the pandemic, is known as coping (Taylor & Stanton, 2007).

### Religious Coping and the RCOPE

Since the 1990s, there has been increased attention to the role of religion in coping with stressors like illnesses and disasters. Kenneth Pargament, a trailblazer in the study of religious coping, has made a distinction between positive religious coping and negative religious coping (Pargament et al., 2000). Challenging circumstances can be taxing on faith because they challenge expectations about the way the world works and thereby may disrupt beliefs about the divine (Aten et al., 2019, p. 598). But research has shown that those who can hold on to positive views about God experience less anxiety and depression and a higher sense of well-being and posttraumatic growth (see for example Aten et al., 2019, p. 605 and Ano & Vasconcelles, 2005). The brief set of questions developed by Pargament and his colleagues to measure positive and negative religious coping has become the most commonly used measure of religious coping (Pargament et al., 2011, p. 72).

Although the theory of religious coping is currently most often used to distinguish positive and negative religious coping and their effects, Pargament’s original theory of religious coping is much broader than this. In *The Psychology of Religion and Coping* (1997), he set out a framework that discusses various functions of religious coping. In a later article, he and his colleagues presented the RCOPE, a measure to assess the full range of religious coping methods in a standardized way (Pargament et al., 2000). This fine-grained analysis of the expressions of religious coping is unfortunately rarely explored in research, mainly because the set of items is very long (Pargament et al., 2005, p. 490; Lehman & Steele, 2020, p. 346).

### Religious Coping in the COVID-19 Pandemic

In the best of circumstances, religion can inspire people to view or handle a difficult situation in a certain way; it can bring solace in challenging circumstances; it has the power to transform suffering. These beneficial effects of religious coping are often mediated by social interactions in a religious community. Belonging to the group of people that form a church can bring social support which may alleviate suffering (Krause et al., 2001). This social support may come from other members but also from clergy. In their sermons, pastors draw on the religious archive for resources to encourage their audiences to look at religion as a source of coping. During the COVID-19 pandemic, this social dimension of the church was considerably restricted by lockdowns and social-distancing measures. Although congregations have attempted to reach out to their members in different ways, the most straightforward way in which the work of the church was continued was by taking worship services online (see for example Capponi & Araújo, 2020 and Parish, 2020).

There has been some research into religious coping during the pandemic. In the USA, negative forms of religious coping were found to be related to a higher degree of anxiety around the coronavirus (DeRosset et al., 2021). In Pakistan, the United Arab Emirates, and Palestine, community members found support in religious practices to cope with the stress and uncertainty of the pandemic (Ali et al., 2021; Mahamid & Bdier, 2021; Thomas & Barbato, 2020). Similar effects have been found in Europe. During these challenging times, people seem to pray more, or at least use search terms related to prayer on Google more (Bentzen, 2020), and also use more religious apps (Camarines & Camarines, 2022) In Italy, the streaming of online masses increased more in areas that were more affected by the pandemic, also suggesting a turn to religion (Alfano et al., 2020).

According to Counted et al. (2022), the role of religious coping could be even more important in vulnerable contexts where other forms of help are lacking. According to their study in Colombia and South Africa, well-being was higher among those with low dispositional hope if they could draw on positive religious coping styles (Counted et al., 2022, p. 78). This article centers on the situation in one such vulnerable context, namely Zambia.

### Zambia and the Reformed Church in Zambia

Zambia went into a relatively strict lockdown early in the pandemic, before the first case was even recorded in the country. From 28 March to 24 May 2020, most of the Christian churches in Zambia stopped their in-person worship services and other group activities, following the directive from the Ministry of Health. The collective church mother bodies, umbrella organizations in which Catholic, Protestant, Evangelical and Pentecostal churches are represented, supported the adherence to this directive (Mwale & Chita, 2022, p. 162f) During this time, pastors in mainly urban areas who had not done so before started live-streaming their services on Facebook or posting them on YouTube (Kroesbergen-Kamps, 2020, p.77f). In this article, the styles of religious coping that pastors in the Reformed Church in Zambia (RCZ) offer to their audiences are investigated.

Zambia is a predominantly Christian country—or even a Christian nation, as it is stated in the preamble to the country’s constitution. The Reformed Church in Zambia is, like the Roman Catholic Church and the Presbyterian churches, one of the traditional mission churches that came to Zambia through missionary activity in the nineteenth century. Newer Christian religious movements like the Seventh-day Adventists and Jehovah’s Witnesses also have a relatively large presence in Zambia. Since the 1980s, there has been a marked growth in Pentecostal churches, both from outside Zambia and originating within Zambia, many of which have elective affinities to international Pentecostal ministries. The Pentecostalization of the Zambian religious landscape is felt by the traditional mission churches as well, with theological debates sometimes leading to divisions. The Reformed Church in Zambia is a church on the crossroads between a traditional Reformed perspective and a more Pentecostal or evangelical view. The diversity present in the church makes it an interesting site for research.

### The Present Study

This study aims to analyze the use of religious coping during the first months of the COVID-19 pandemic in Zambia. In particular, it investigates the methods of religious coping provided by pastors in live-streamed services, since in these first months other forms of pastoral care were not accessible to congregations because of strict lockdown regulations. The results are analyzed using the framework of religious coping developed by Kenneth Pargament and interpreted in the context of African Christianity. This study is one of the few to use the complete range of religious coping methods that were described and measured by Pargament (1997, 2000). The insights gained from this analysis shed light on the under-researched topic of religious coping in non-Western, African contexts. The results may also identify forms of religious coping not captured in the current literature.

## Method

### Sample

Through the researcher’s extensive network of pastors in the RCZ as well as additional inquiries, 36 RCZ pastors and congregations who used Facebook or YouTube to reach their audiences between March and September 2020 were identified. Over 200 services held in this period were transcribed. However, it was only in the services that were broadcast during the period of lockdown from 29 March to 24 May that there was a significant amount of attention to the pandemic; therefore, this article focuses on that period. Some pastors live-streamed only one service or posted videos only sporadically, for instance, because of technical difficulties, lack of funds, or because their congregations did not have access to the online services.

For this article, a group of 20 pastors who each preached online four or more times during the period of lockdown was selected. All of these pastors were male and from urban or peri-urban areas. Together, they contributed 134 online services. Of these services, the relevant parts that addressed the current situation were transcribed in a clean verbatim style. Other parts of the service, such as biblical exegesis or applications on topics that had nothing to do with the pandemic, were summarized in some detail, but not captured verbatim.

This research was approved by the Research Ethics Committee of the Faculty of Theology and Religion at the University of Pretoria. For interviews with the pastors, informed consent was obtained. All names of the pastors in this article are pseudonyms.

### Choice of Content

In this time of crisis, pastors in the RCZ felt the need to encourage their congregations and offer them ways to cope with the difficult circumstances in which they found themselves (Kroesbergen-Kamps, 2020, p. 82). To see what particular styles of religious coping pastors in the Reformed Church in Zambia offer their audiences, the sermons of the 134 services were analyzed. Prayer can be important in religious coping as well, or may even be a form of religious coping, but in this article, the focus lies on the more analytical and reflective utterances, the sermons.

### Application of the Religious Coping Framework

The full RCOPE measures five different functions of religious coping through 21 subscales that tap into the degree to which people make use of different religious coping methods. Each subscale is measured by multiple items which have the form of statements about religious coping.^1^ The functions of religious coping are theoretically grounded in a functional analysis of religion (Pargament et al., 2000, p. 520–521). The five functions are:

- **Religious Methods of Coping to Find Meaning**
- **Religious Methods of Coping to Gain Control**
- **Religious Methods of Coping to Gain Comfort and Closeness to God**
- **Religious Methods of Coping to Gain Intimacy with Others and Closeness to God**
- **Religious Methods of Coping to Achieve a Life Transformation**

These functions are further divided into several subscales, which are measured by items which were developed by Pargament et al. from existing scales, clinical literature as well as interviews (2000, p. 527). For example, the function **Religious Methods of Coping to Find Meaning** contains the following subscales:

- *Benevolent Religious Reappraisal*
- *Punishing God Reappraisal*
- *Demonic Reappraisal*
- *Reappraisal of God’s Powers*

The items in the subscale of *Benevolent Religious Reappraisal* are the following:

- Saw my situation as part of God’s plan
- Tried to find a lesson from God in the event
- Tried to see how God might be trying to strengthen me in this situation
- Thought that the event might bring me closer to God
- Tried to see how the situation could be beneficial spiritually

The full RCOPE gives a comprehensive measurement of all five functions of religious coping, but takes longer to administer and is therefore rarely used in empirical studies. For this research, however, the fine-grained framework of the full RCOPE was used to explore the ways of religious coping that are addressed, encouraged, and discouraged by pastors in the RCZ through their sermons.

### Coding Procedure

Studies that use the RCOPE or the Brief RCOPE are almost exclusively quantitative studies because the tool developed by Pargament et al. comes in the form of items that can easily be added to a survey. In this case, however, the items on the RCOPE will be applied in a more qualitative way to the Zambian sermons. To that end, sections (most often two or three sentences) from the sermons were coded with labels based on the items on the RCOPE. In many instances, the pastors would almost literally say a phrase that appears as one of the items in the RCOPE. For example, one of the items of *Seeking Spiritual Support*, a subscale in the function of **Religious Methods of Coping to Gain Comfort and Closeness to God**, is, Trusted that God was with me. One of the pastors, Pastor Ganizani, said the following in a sermon: “Our God is ever-present because he has promised never to leave us nor to forsake us, but to be with us in every situation, even in this situation we are passing through.” In this passage, the pastor is encouraging his congregation to trust that God is with them, and therefore, it was labeled as such.

In some cases, the pastor did not use the exact item noted in the RCOPE but expressed a sentiment that clearly belonged in a certain subscale. These have been coded as *Other*, discussed with a colleague, and if there was agreement placed within that subscale. For instance, to stay within the same subscale of *Seeking Spiritual Support*, in one sermon, Pastor James said, “We see that God, as all-powerful God, is concerned for all his people, regardless of their circumstances. He is also concerned about you, about our nations, about the church in Zambia, about the happenings in the world, and about what surrounds you right now.” Although he does not say literally that God is with people or at their side, this is the sentiment that speaks from this passage. It was therefore coded as *Seeking Spiritual Support*—Other.

Sometimes, the pastors expressed methods of religious coping that had no clear equivalent to the items of the RCOPE. These were again discussed with a colleague and placed within the subscale or function of religious coping that fitted best. An example of a method of religious coping that is—surprisingly—absent in the RCOPE is the reference to heaven or eternal life. Pastor Patrick, for example, says, “World over some have died already with the coronavirus, but the message I bring to the families who have lost beloved ones is: do not despair, because Jesus, and by his grace, he gives us hope for eternal life. Those who believe in God, it doesn’t matter what type of death takes them. One encouragement we have is that we are going to meet in glory.” A belief in the afterlife, in better things to come, is a way to alleviate the challenges of today; a method of religious coping. Within the RCOPE, no item or subscale comes close to this sentiment, but it seems to fit best within the function of **Religious Methods of Coping to Gain Comfort and Closeness to God**, and therefore, it was labeled as a new subscale within that function: *Faith in Eternal Life*. A full list of the functions and subscales of the RCOPE, including additions based on this research, can be found in Appendix 1.

## Results

Perhaps unsurprisingly, all pastors make mention of the pandemic at some point in their services. However, it is not a topic that gets attention all the time. Especially during the feast of Easter and toward the end of the period of lockdown, the pandemic and its effects were mentioned less frequently than in the beginning. A little over a third of the sermons have the pandemic as their main theme. In another 40%, the pandemic is mentioned, but as one of several examples or mainly in the prayers. The remaining 20% of the services mention the pandemic only as a sidenote, or not at all.

In this discussion of the results of my analysis, the functions, subscales, and items of religious coping that are the most prevalent in the Zambian sermons will be highlighted. Appendix 1 gives an overview of all of the methods of religious coping used in the sermons.

### Seeking Spiritual Support

There does not seem to be a clear method or set of methods of religious coping that is shared by all pastors or used in all sermons. The subscale that is mentioned most often is *Seeking Spiritual Support* (Table 1).

**Table 1:** Seeking spiritual support

Religious Methods of Coping to Gain Comfort and Closeness to God	In no. of sermons (N = 134)	% of pastors using it at least once (N = 20)
*Seeking Spiritual Support*—Totals:	49	90
Per item:		
Sought God’s love and care	2	10
Trusted that God would be by my side	4	15
Looked to God for strength, support, and guidance	14	40
Trusted that God was with me	14	45
Sought comfort from God	2	10
Added:		
Other	7	30
Trusted that God is in control	10	40
Trusted that God is greater	12	40
Trusted that in God we are victorious	12	35

Of the pastors, 90% urge their audience to seek spiritual support at least in one sermon. It is often used not only in the sermons that have the pandemic as their main theme but also in sermons that deal with other issues, as a sort of baseline of faith in these challenging times. Phrases like Trust that God is with us, or Look to God for strength, support, and guidance, which are on the RCOPE questionnaire, are often mentioned. Some items that are not on the RCOPE, but that communicate the same sentiment, were added as well, namely, God is greater, In God we are victorious, and God is in control. Each one of these items is mentioned in 10 to 12 sermons. In the following section, representative examples of each of them and a motivation for their placement under the heading of *Seeking Spiritual Support* are presented.

The first item is that God is in control. Pastor Mwaaza, for example, states:

The big question after this pandemic is: what will life look like? Will the economies be repairable at all? Or what will become of our families? Well, we can’t give very good answers to that. But one thing we know is that God will always be in control. Therefore, blessed be the name of the Lord. God is still on the front. He reigns and he hasn’t been surprised by what has taken place.

In the end, it is God who is in control of the pandemic. The second item, God is greater, explains why God is in this position of control. Pastor Benjamin says:

God is greater than the coronavirus, so we need to magnify him. The Lord is fighting for us. We may not see him physically, but this God is fighting for us. He is a mightier God and greater God than our enemy. That is the God that we serve. It doesn’t matter how gloomy the situation may be, this God remains God forever and ever and ever and ever.

God is greater than the pandemic the world is experiencing. The pandemic may pose a challenge to us humans, but not to God. The image of God fighting for us that Pastor Benjamin uses comes back even stronger in the third item, In God we are victorious. Pastor Timothy says:

Jesus is our representative in this battle. He is representing us, meaning the victory of Jesus becomes our victory. We have a great conqueror who is coming with the message, “Do not worry, do not fear, but peace be with you.” It is over. Jesus has fought a good fight onto the benefit of every one of us. We need to be encouraged in our faith because of the nail marks in the hands of our savior Jesus Christ. It is well with your life, with your family, with your business, because Jesus has conquered.

Because God is greater, his victory over the pandemic is already ensured, and if God is victorious, it means that his people join in that victory. Pastor Timothy does not even speak about this victory as something that lies ahead in the future; it is already done.

The three items that were added to the subscale of *Seeking Spiritual Support* are conceptually close to each other. God is in control because he is greater than the coronavirus. He will provide the victory, even if our situation right now is still dire and confusing. The subscale that best fits this complex of ideas is that of *Seeking Spiritual Support*. God is our spiritual support, the Zambian pastors seem to say. We can rely on him, trust him, accept his guidance, and be comforted by the thought that he will be victorious.

It may seem strange to place the idea that God is in control in the subscale of *Seeking Spiritual Support* within the function of **Religious Methods of Coping to Gain Comfort and Closeness to God** when there is on the RCOPE also a whole function devoted to control: **Religious Means of Coping to Gain Control**. In the cases of these items, it is assumed that they are meant to comfort audiences more than give them a sense of control. That being said, the function of **Religious Means of Coping to Gain Control** is important in the Zambian services as well.

### Religious Means of Coping to Gain Control

The religious methods of coping to gain control within the RCOPE differ from each other in their emphasis on human or divine agency. On the far end of the human spectrum, there is the subscale of *Self-Directing Religious Coping*, in which no help is sought from God at all. On the other of the spectrum, on the side of divine agency, lies the subscale of *Passive Religious Deferral*, in which God is believed to bring the solution to the challenge while humans patiently wait. Between those two extremes lie different levels of cooperation between humans and God, from *Collaborative Religious Coping* to *Active Religious Surrender* to *Pleading for Direct Intercession* or divine intervention. In all of these, humans and God both are expected to take some actions. Table 2 shows the results in the Zambian sermons.

**Table 2:** Religious methods of coping to gain control

Religious Methods of Coping to Gain Control	In no. of sermons (N = 134)	% of pastors using it at least once (N = 20)
*Collaborative Religious Coping*	8	20
*Active Religious Surrender*	14	45
*Passive Religious Deferral*	12	40
*Pleading for Direct Intercession*	10	35
*Self-Directing Religious Coping*	7	25
*Added: Instrumental Expression of Firm Faith*	20	65
*Added: Instrumental Positive Confession*	8	35

In the Zambian sermons, *Self-Directing Religious Coping* is outrightly rejected. If it is mentioned at all, it is negatively. Pastor Samuel, for example, says:

One person said, “Prayer is not the answer to everything. There are things that you need to pray about, but not COVID-19. Leave it to the scientists, leave it to the government.” But as far as we are concerned as children of God, God wants to get involved in every matter that concerns us. Every issue of our life, he wants to get involved.

Self-reliance in the face of the pandemic, or even reliance on medical sciences or the government, is portrayed as a temptation. Challenges should routinely be brought before God, the pastors say.

Some Zambian pastors encourage their audiences to wait passively for God to make things right: *Passive Religious Deferral*. This stance is understandable given the emphasis on God’s power in the popular items about spiritual support. Most common, however, are the positions in the two extremes of human and divine agency. Especially the more instrumental subscales, *Active Religious Surrender* and *Pleading for Direct Intercession*, in which Christians need to do something before God will help them, are used often in the sermons. A few examples follow.

Pastor Dalitso says:

Let us observe social, others say physical, distancing. Let us observe the washing of the hands, sanitizing, but also the wearing of the mask. Let us have faith that we can even do something about it. We do our part and God will do his part as well.

In the RCOPE, this is a clear example of *Active Religious Surrender*. Humans need to do sensible things like keeping distance, sanitizing, and wearing masks, but after that is done, God will offer healing and protection which are still necessary. Human control only reaches so far; beyond that, everything lies in God’s hands.

The actions that are necessary to ensure God’s help can also seem less reasonable from a secular point of view. Prayer as a means of gaining religious control—labeled as *Pleading for Direct Intercession* by Pargament et al.—works on the premise that if Christians ask, God will give them what they need. Pastor Patrick says:

These are moments that you need to go on your knees, if you can, to begin raising up your voice to this living God, that he may intervene in this situation.

God’s intervention is needed, and if his people pray, he will heed their calls. But prayer is not the only action that may drive God into action. These other actions are not represented on the RCOPE, but they occur often enough in the Zambian sermons to add them to the subscales within the broader function of **Religious Means of Coping to Gain Control**.

The first of these alternative human actions to ensure God’s intervention is to stand firm in faith, which I have labeled as *Instrumental Expression of Firm Faith*. This is mentioned in 20 different sermons, the second-most used subscale within the Zambian sermons. It is mentioned by 65% of the pastors, again, the second-highest amount after the subscale of *Seeking Spiritual Support*. Pastor Timothy expresses it very clearly:

We must believe in order to receive from God. God is pleased only with our faith. Even in coronavirus, we need not to doubt the victory of Jesus Christ. We have a warrior who has conquered, his name is Jesus Christ. The only way to come out as conquerors, we need to believe what God has done for us.

A firm belief in what God can do is presented as a prerequisite for his help. Here, the assurance that God has the victory is not just something that brings comfort, as it was within the subscale of *Seeking Spiritual Support*. Only those who believe that God will help them will be saved.

Related to the emphasis on a firm belief is also the relatively high number of pastors that reject *Spiritual Discontent* and a *Reappraisal of God’s Powers*, two subscales on the RCOPE that are only mentioned negatively (see Appendix 1). According to research, negative life events, such as the COVID pandemic, can lead to religious struggles, doubt, and loss of faith (Captari et al., 2022). In the Zambian sermons, no room is given for doubt. A clear sign of this belief is the positive confession that what God may do for us has already happened. Pastor Michael explains this:

What is it that God wants us to do amidst this pandemic? We should declare life, not death, over our situations. We should not be confessing how depressing the situation is. Say that it is well. When we say that, it’s because we understand that the tongue that God gave to us was given power by God himself to bring life or death. So, when you speak life, life comes. When you speak death, death comes as well. So, choose to speak life and resist the temptation to speak death. Don’t share, even on social media, depressing messages. Say, “It is well.”

Speech has the power to attract positive or negative things. Someone who speaks positively, this is the idea behind positive confession, thereby claims the positive things that God has in store for them. This instrumental way of speaking good things into one’s life—labeled in Table 2 as the subscale of *Instrumental Positive Confession*—is not used as often as the effects of firm faith, but it is a current that is not uncommon in the more Pentecostal parts of the Reformed Church in Zambia.

### Benevolent Religious Reappraisal

After the promise and comfort of *Seeking Spiritual Support*, the focus on human actions to bring forth God’s help and thereby control the challenging situation expressed in the function of **Religious Methods of Coping to Gain Control** is the most used means of religious coping. The third-most high-scoring subscale on the RCOPE is the *Benevolent Religious Reappraisal*, in which the challenging circumstance is imbued with meaning by making it part of God’s plan or attempting to see how it can be beneficial, for example, because it may teach one a lesson (Table 3).

**Table 3:** Benevolent Religious Reappraisal

Religious methods of coping to find meaning	In no. of sermons (N = 134)	% of pastors using it at least once (N = 20)
*Benevolent religious reappraisal*—Totals:	19	50
Per item:		
Saw my situation as part of God’s plan	7	25
Tried to find a lesson from God in the event	5	25
Tried to see how God might be trying to strengthen me in this situation	0	0
Thought that the event might bring me closer to God	1	5
Tried to see how the situation could be beneficial spiritually	7	35
Other	7	25
*Added: Reappraisal of World History (Apocalypse)*	15	55

Half of the pastors use the subscale of *Benevolent Religious Reappraisal* as a means of religious coping at one point during a sermon. It is especially prevalent within sermons that have the pandemic as their main focus: in 84% of the cases in which it is mentioned, it is within a corona sermon.

In the following, a few examples of this *Benevolent Religious Reappraisal* are presented. The main idea here is that there is a purpose behind the suffering that the world is going through. It is part of God’s plan, as pastor Mwaaza explains:

Look at what the world is experiencing right now. Very humbling. How can one nation experience more than 2,000 deaths out of the COVID-19 in 24 hours? That’s an experience. It doesn’t mean that God is surprised by this. He is aware of what is happening. Ultimately, the plan is in his hands. That is humbling.

This fits with the RCOPE item: Saw my situation as part of God’s plan. God’s plan is a plan for our benefit. Pastor Mwaaza emphasizes that the pandemic encourages us to learn something—summarized in the RCOPE as the item: Tried to find a lesson from God in the event:

Let this be an opportunity for you to renew your strength, to grow in God, to grow in your faith. Probably the time of seclusion, to be alone in the presence of God, has lessons.

Pastor Vickson wonders what these lessons might be:

Who knows, maybe he wants us to build better businesses, better relationships than ever before. Who knows, maybe God wants us to come out better Christians than we were. We have understood the value of fellowship by just being absent from each other.

Pastor Ganizani mentions another lesson:

It is not that the Lord has stopped caring, but he allows us to pass through certain situations in life so that our faith can be tested and to show us that he is in control.

Both Pastor Vickson and Pastor Ganizani fit with the RCOPE item of Tried to see how the situation could be beneficial spiritually. According to Pastor Ganizani, the coronavirus is a test of our faith. It will teach us that it is God who is in control of our fate.

Again, all of these items center on the idea that God is in control of this challenge humankind is passing through. It is his plan, his lesson that his people have to learn that gives the pandemic a positive meaning.

A final subscale that is often mentioned in the Zambian sermons is also related to God’s plan with the world. Some pastors do not just seek personal lessons in the pandemic but place it in a narrative about world history. They see the pandemic as a possible sign that the last days of the Apocalypse are drawing near, a subscale that is added as *Reappraisal of World History (Apocalypse)* An example of a nuanced discussion of the pandemic and the end of times comes from Pastor Michael:

Through this pandemic, God is just giving the world a wake-up call. He is reminding us that the world as we know it one day will come to an end. […] Sometimes, when you are living in this world and everything is okay, we tend to deceive ourselves, thinking that things will always be like this. No, no, no, no, no. One day this world as we know it will not be here. It will be no more. The world will be no more. When the trumpet of the Lord shall sound and the dead shall be able to be resurrected, it will be a time for judgment. So, it means that it is time for us to prepare. Through this pandemic, God is reminding us that judgment is coming. You need to prepare to meet your God, your Maker, your Creator, on the Judgment Day.

Most pastors are, like Pastor Michael, very careful not to explicitly predict anything, but it is telling that more than half of the pastors at least feel the need to mention the possibility that the pandemic is related to the Apocalypse. A similar focus on COVID-19 as a fulfilment of end-time prophecies is mentioned by Sonene Nyawo in an analysis of Christian understandings of the pandemic in Eswatini (2022, p. 145f).

### Under-represented Categories

As interesting as the styles of religious coping that the pastors encourage their audiences to take on are the things that are missing. Thus far, the first three functions contained in the RCOPE have been discussed, namely **Religious Methods of Coping to Find Meaning**, **Religious Methods of Coping to Gain Control**, and **Religious Methods of Coping to Gain Comfort and Closeness to God**. All of these were well-represented in the Zambian sermons.

Within the function of **Religious Methods of Coping to Find Meaning**, two subscales are surprisingly absent, namely *Punishing God Reappraisal* and *Demonic Reappraisal*. The former explains misfortune as a divine punishment, while the latter sees it as caused by forces of evil, such as the devil. Both subscales are mentioned in a small number of sermons but are not presented as important strategies to cope with the COVID-19 pandemic. This is surprising since for example Nyawo in an analysis of Christian responses to the pandemic in Eswatini found that “COVID is God’s punishment for sin” and “COVID is spiritual warfare between God and Satan” were the most prominent understandings (2022, p. 143f). Whether this lack of importance in the Zambian sermons is due to the character of Christianity in the RCZ or to the choices make by pastors for their sermons is a question for further investigation.

Besides the three mentioned functions, I RCOPE adds two more functions, namely **Religious Methods of Coping to Gain Intimacy with Others and Closeness to God** and **Religious Methods of Coping to Achieve a Life Transformation**. In the Zambian sermons, both of these categories are under-represented.

The function of **Religious Methods of Coping to Gain Intimacy with Others and Closeness to God** measures horizontal forms of religious coping, for example *Seeking Support from Clergy or Members* and *Religious Helping*. In another publication (Kroesbergen-Kamps, 2020), the author has shown that an interesting feature of the Zambian sermons is that they have a vertical rather than a horizontal orientation: they focus on the relationship with God much more than on relations with other human beings such as members of the congregation. The low score on this function is therefore not unsurprising.

The function of **Religious Methods of Coping to Achieve a Life Transformation** is also mentioned in very few of the Zambian sermons. This will be discussed in more detail in the next section.

## Analysis of Results

### Conservation and Reconstruction Rather Than Transformation

When difficult circumstances arise, the first response is often to be conservative. Among the means of religious coping that Kenneth Pargament mentions as mechanisms used to sustain a way of life are religious perseverance and spiritual support (Pargament, 1997, p. 201). The emphasis on the Instrumental Expression of Firm Faith, which is an important element in the Zambian sermons, can be read as a way to get through difficult circumstances through perseverance. Furthermore, *Seeking Spiritual support* is the most important means of religious coping used in all of the sermons. Audiences are reminded of God’s love and care, and that God, in the end, is in control.

When wholesale conservation is impossible, a reconstruction of beliefs may be necessary, for instance through religious reframing (Pargament, 1997, p. 222). The attempt to give the pandemic a religious meaning in the subscale of *Benevolent Religious Reappraisal* is an example of such religious reframing. Sometimes, conservation and even reconstruction are not possible, and the only way to retain religious significance may be through a transformation of beliefs (Pargament, 1997, p. 110, 235). In the sermons discussed in this article, the function of **Religious Methods of Coping to Achieve a Life Transformation** is almost absent. It may be that, at a later stage in the progression of the pandemic, the function of religious transformation on the RCOPE would gain greater significance. In the sermons analyzed for this article, however, the theme of transformation is not yet important.

### The Statement ‘God is in Control’ in Lived Religion

In the discussion of the results, it has become clear that many of the Zambian statements surrounding the idea that God is in control are missing from the RCOPE. This might suggest that this is a typical African response to a crisis. However, most of the Zambian additions to the RCOPE are not so much specifically African as evangelical or, in some cases, Pentecostal in nature.

The growth of Christianity in the global south has been widely noted and commented upon (see for example Jenkins, 2011). The type of religion that is most successful in Africa emphasizes the active intervention of good as well as evil spiritual forces in this world. This fits with an African understanding in which misfortune and achievements are both often related to either opposition or support from the spiritual realm (see for example Meyer, 2004). If a crisis like the pandemic strikes, this is naturally related to disturbances in the relationship with the world of spiritual powers. In itself, however, the idea that God can intervene in this world to bring about healing and other blessings is not limited to Africa and is not uncommon in the USA either.

In the Zambian sermons, the idea that God is in control seems to be behind the most used forms of religious coping. “God is in control” is a comforting expression that ensures audiences that spiritual support is there for them. It also gives meaning to the pandemic: human suffering is not random, without purpose, but it is part of God’s plan, possibly even significant in terms of the end of the world, and may, in the end, be beneficial to those who can learn from it. The sermons also give indications as to how to access and employ God’s control for the audience’s benefit. If a Christian believes firmly, asks for God’s intervention, and speaks positively, God will come to his rescue, according to the sermons.

That God is in control fits with another striking statement that comes back in several sermons, namely that humans are not in control and should not be trusted to solve the issue of the pandemic. Science, doctors, and the government all seem to be confused and are not able to prevent the pandemic. All these human efforts are disappointing, explains Pastor Madalitso, but there is another to look to:

Doctors may fail, but Jesus will never fail. Professors may fail, but Jesus will never fail. Pastors, bishops, prophets may fail, but Jesus will never fail. Man may fail, but Jesus will never fail because he is a great God, he is a mighty God, he is the king of glory.

It may be that the disappointment in human efforts leads to a focus on God’s control, or it may be that a focus on God’s control leads to mistrust of what humans are capable of. In any case, God’s control and human self-reliance seem to be inversely related.

During the pandemic, African churches have in opinion pieces been accused of ‘spiritualizing the virus’ by focusing on spiritual causes of and support against the coronavirus instead of encouraging people to seek practical means of protection in this world (see, for example, Parsitau, 2020 and Asamoah-Gyadu, 2020). On a surface level, this interpretation of what African churches do fits with Robin Horton’s theory of religion. According to Horton (1993), religion has two dimensions, namely communion with God and providing explanation, prediction, and control of worldly circumstances. Spiritualizing the coronavirus or other problems seems to be an example of that second dimension of religion according to Horton: through religion, the coronavirus is explained, its course predicted, and means of controlling it are offered.

This interpretation, however, fails to do justice to the role of religion in people’s lived experiences. Horton’s dimension of explanation, prediction, and control can easily be read as if religion is a kind of alternative scientific method. Within lived religion, however, the explanations, predictions, and means of control offered by religion are not treated in the same way as scientific thought. As the Wittgensteinian philosopher of religion Hermen Kroesbergen (2020) argues, religious explanations have a personal and relational validity, rather than the general, universal validity of scientific explanations. Instead of taking religion as an alternative provider of a sort of scientific explanation, prediction, and control, a closer look at the way religion is lived shows that religious explanations and means of control work very differently. Religion speaks of control where there is no control and is an explanation where human explanations are failing (Kroesbergen, 2019, pp. 47–58). Take for example the expression “God only knows.” This statement appears to presuppose the existence of an all-knowing God. However, if one looks at how this statement is used and when it is uttered, it becomes clear that it is not said to make a truth-claim about the nature of God, but as a way to express that we human beings do not know.

Many of the statements in the Zambian sermons can be read similarly. Emphasizing that God is in control is comforting because it expresses unease about the fact that humans are not in control. Stating that the pandemic is part of God’s plan is an indication that humans have not been able to predict it. From this perspective, the statements in the sermons that emphasize that humans are prone to failure and cannot be depended on make more sense as well. In this time in which so many certainties are shattered, religion provides a language to speak about contingencies. Saying that God is in control does not necessarily spiritualize the virus. Rather, it expresses the conviction that there are situations that cannot be controlled by humans, and these should be related to the domain of God, not because God will provide an immediate solution, but because this is the way to express the experience of contingency.

### ‘God is in Control’ and Adherence to Health Measures

If the statement that God is in control is viewed as an expression of contingency rather than a way to control circumstances, it is understandable that most pastors urge their audiences to have faith in God’s control over the situation while at the same time encouraging them to wash their hands, wear facemasks and limit unnecessary movements. Pastor Madalitso, for example, who was quoted above saying that not doctors, not professors, not pastors, nor man in general, can help but only God, also makes the following statement:

Please stay safe. Observe all the necessary precautions that we need to observe in order to prevent contracting the COVID-19 virus as well as spreading it. Wash your hands frequently. Be at home. Unless otherwise, make sure that we stay at home.

This is not to say that there are no risks in the way people relate God to the pandemic. Some forms of religious meaning-making can be misguided if they are used to the exclusion of other forms of dealing with a crisis (Pargament, 1997, pp. 326f). Pargament also sees an overemphasis on religious resources and actions such as prayer as a form of misguided religious coping. If a situation is to some extent controllable through this worldly actions, religious actions should not preclude what someone can practically do (Pargament, 1997, pp. 328f). If pastors would downplay the importance of health measures in favor of ritual actions such as prayer or fasting, that would be misguided. But the idea that God is in control in itself does not necessarily exclude other, more practical, responses to the pandemic. It functions in a different register than these science-based responses.

## Limitations

The method of qualitative analysis of the sermons using the quantitative tool of the RCOPE used in this article has benefits as well as some limitations. It is important to consider that this research does not measure the method of coping of any particular group. The audience of the sermons can take or leave the encouragement of their pastors, and even the pastors may be giving advice that they themselves are unable to follow. It is possible that a pastor, when asked to fill out an RCOPE questionnaire, will, for instance, strongly agree with the statement that he has questioned the power of God in this crisis, even though in his sermons he will reassure his congregation that God is in control. What this study shows is which methods of religious coping are mediated preferentially by pastors to their audiences, thereby sanctioning them in the context of the Reformed Church in Zambia.

This being a qualitative study with a relatively small sample size of sermons, statistical analysis of for example correlations between subscales of the RCOPE was not possible. The research relies heavily on the way in which the statements in sermons were coded according to the RCOPE. The main work of coding was done by one researcher. This means that there is a risk that preconceived ideas or biases of the coder could influence the results. To mitigate this risk, as declared in the section on methods, when in doubt the attribution of statements to items, subscales and functions within the RCOPE was discussed with others.

## Conclusion

This study used a quantitative tool, the RCOPE, qualitatively, interpreting the words of Zambian pastors in their sermons. Qualitative research is a good way to explore new themes or new settings. Some authors have mentioned that the RCOPE has mainly been used in a Western context, and a qualitative approach like this can uncover religious coping themes that are not integrated into the RCOPE because of its cultural and geographical bias (cf. Pargament et al., 2005, p. 490; Kwilecki, 2004, p. 484). An important yield of this research is the uncovering of several subscales and items of religious coping that are omitted in the RCOPE, such as the underlying view that God is in control and the power of positive statements. These new subscales are not so much in line with a specifically African worldview, but with a more evangelical or Pentecostal variety of Christianity that is under-represented in the RCOPE.

Because the selected passages from the sermon are generally longer than the brief items included on the RCOPE, this study has given a more detailed view of what people may mean when they agree with these statements. In this case, the Zambian emphasis on God’s control of the situation surrounding the pandemic could be explained not as magical thinking or an alternative to scientific responses to disease, but as belonging to a different, religious register of speaking about contingencies.

## Appendix 1

**Table:**

	In no. of sermons (N = 134)	% of pastors using it at least once (N = 20)	% of mentions in corona sermons*
**Subscales and added items within the function of Religious Methods of Coping to Find Meaning**			
*Benevolent Religious Reappraisal* (Added item: Other)	19	50	84
*Punishing God Reappraisal* (Added item: Other)	4	20	75
*Demonic Reappraisal* (Added item: Other)	2	5	50
*Reappraisal of God’s powers* (Added item: Other)	10	35	70
Added subscale:* Reappraisal of World History (Apocalypse)*	15	55	40
**Subscales and added items within the function of Religious Methods of Coping to Gain Control**			
*Collaborative Religious Coping* (Added item: Other)	8	20	88
*Active Religious Surrender* (Added item: Other)	14	45	86
*Passive Religious Deferral* (Added item: Other)	12	40	75
*Pleading for Direct Intercession* (Added item: Other)	10	35	50
*Self-Directing Religious Coping* (Added item: Other)	7	25	86
Added subscale: *Instrumental Positive Confession*	8	35	75
Added subscale: *Instrumental Expression of Firm Faith*	20	65	55
**Subscales and added items within the function of Religious Methods of Coping to Gain Comfort and Closeness to God**			
*Seeking Spiritual Support* (Added item: Other*,* Trusted that God is in control*,* Trusted that God is greater*,* Trusted that in God we are victorious)	49	90	57
*Religious Focus* (Added item: Other)	2	10	100
*Religious Purification* (Added item: Tried to live a pure life)	11	45	36
*Spiritual Connection* (Added item: Other)	13	45	69
*Spiritual Discontent* (Added item: Other)	13	45	85
*Marking Religious Boundaries* (Added item: Other)	13	45	54
Added subscale: *Faith in Eternal Life*	12	40	33
**Subscales and added items within the function of Religious Methods of Coping to Gain Intimacy with Others and Closeness to God**			
*Seeking Support from Clergy or Members* (Added item: Other)	2	5	100
*Religious Helping* (Added item: Practical help)	9	30	67
*Interpersonal Religious Discontent* (Added item: Other)	2	10	0
**Subscales and added items within the function of Religious Methods of Coping to Achieve a Life Transformation**			
*Seeking Religious Direction*	0	0	0
*Religious Conversion* (Added item: Other)	5	20	60
*Religious Forgiving* (Added item: Other)	1	5	100
Added subscale: *Planning for the Future*	8	25	63

*Of the total number of sermons, a third have the pandemic as their main theme. These I call the corona sermons. In other sermons, the pandemic is mentioned less frequently or not at all. But elements of the RCOPE may still show up in these non-corona sermons. The third column shows whether the mentions come from corona sermons or sermons in which corona is not the main theme. *Benevolent Religious Reappraisal*, for example, is a subscale that is mentioned mainly in corona sermons: 84% of the mentions of this subscale happen in a sermon that has corona as its main topic. On the other hand, *Seeking Spiritual Support* is a topic that is mentioned both in corona sermons and in non-corona sermons. In 57% of the cases, it is mentioned in a corona sermon, which means that in 43% of the cases in which pastors draw on the topic of spiritual support, this is in a sermon in which the pandemic is not the main theme. Interesting results in this column are alluded to in the text of the article

## Footnotes

1 In the text of this article, the functions are printed in bold, subscales in italics, and the individual items within each subscale of the RCOPE are underlined. In this way, the different **functions**, *subscales,* and items will be easy to recognize.
